# Synergy between Cell Surface Glycosidases and Glycan-Binding Proteins Dictates the Utilization of Specific Beta(1,3)-Glucans by Human Gut *Bacteroides*

**DOI:** 10.1128/mBio.00095-20

**Published:** 2020-04-07

**Authors:** Guillaume Déjean, Kazune Tamura, Adriana Cabrera, Namrata Jain, Nicholas A. Pudlo, Gabriel Pereira, Alexander Holm Viborg, Filip Van Petegem, Eric C. Martens, Harry Brumer

**Affiliations:** a Michael Smith Laboratories, University of British Columbia, Vancouver, British Columbia, Canada; b Department of Biochemistry and Molecular Biology, University of British Columbia, Vancouver, British Columbia, Canada; c Department of Chemistry, University of British Columbia, Vancouver, British Columbia, Canada; d Department of Microbiology and Immunology, University of Michigan Medical School, Ann Arbor, Michigan, USA; e Department of Botany, University of British Columbia, Vancouver, British Columbia, Canada; Brigham and Women's Hospital/Harvard Medical School

**Keywords:** *Bacteroides*, *Bacteroidetes*, dietary fiber, glycan-binding protein, glycoside hydrolase, polysaccharide utilization locus, polysaccharides

## Abstract

The human gut microbiota (HGM) has far-reaching impacts on human health and nutrition, which are fueled primarily by the metabolism of otherwise indigestible complex carbohydrates commonly known as dietary fiber. However, the molecular basis of the ability of individual taxa of the HGM to address specific dietary glycan structures remains largely unclear. In particular, the utilization of β(1,3)-glucans, which are widespread in the human diet as yeast, seaweed, and plant cell walls, had not previously been resolved. Through a systems-based approach, here we show that the symbiont Bacteroides uniformis deploys a single, exemplar polysaccharide utilization locus (PUL) to access yeast β(1,3)-glucan, brown seaweed β(1,3)-glucan (laminarin), and cereal mixed-linkage β(1,3)/β(1,4)-glucan. Combined biochemical, enzymatic, and structural analysis of PUL-encoded glycoside hydrolases (GHs) and surface glycan-binding proteins (SGBPs) illuminates a concerted molecular system by which B. uniformis recognizes and saccharifies these distinct β-glucans. Strikingly, the functional characterization of homologous β(1,3)-glucan utilization loci (1,3GUL) in other *Bacteroides* further demonstrated that the ability of individual taxa to utilize β(1,3)-glucan variants and/or β(1,3)/β(1,4)-glucans arises combinatorially from the individual specificities of SGBPs and GHs at the cell surface, which feed corresponding signals to periplasmic hybrid two-component sensors (HTCSs) via TonB-dependent transporters (TBDTs). These data reveal the importance of cooperativity in the adaptive evolution of GH and SGBP cohorts to address individual polysaccharide structures. We anticipate that this fine-grained knowledge of PUL function will inform metabolic network analysis and proactive manipulation of the HGM. Indeed, a survey of 2,441 public human metagenomes revealed the international, yet individual-specific, distribution of each 1,3GUL.

## Abstract (precis)

*Bacteroidetes* are a dominant phylum of the human gut microbiota (HGM) that target otherwise indigestible dietary fiber with an arsenal of polysaccharide utilization loci (PULs), each of which is dedicated to the utilization of a specific complex carbohydrate. Here, we provide novel insight into this paradigm through functional characterization of homologous PULs from three autochthonous *Bacteroides* species, which target the family of dietary β(1,3)-glucans. Through detailed biochemical and protein structural analysis, we observed an unexpected diversity in the substrate specificity of PUL glycosidases and glycan-binding proteins with regard to β(1,3)-glucan linkage and branching patterns. In combination, these individual enzyme and protein specificities support taxon-specific growth on individual β(1,3)-glucans. This detailed metabolic insight, together with a comprehensive survey of individual 1,3GULs across human populations, further expands the fundamental roadmap of the HGM, with potential application to the future development of microbial intervention therapies.

## INTRODUCTION

The human gut microbiota (HGM) is a complex community that underpins our nutrition and overall well-being (1, 2) yet is also associated with some diseases (3–6), depending on its particular composition and physiology. A key challenge in manipulating HGM dynamics toward healthful outcomes is a limited understanding of the ecological forces that shape this community within individuals (7, 8). The catabolism of complex dietary carbohydrates is a key driver of HGM structure and metabolic function (9, 10). Thus, resolving a detailed roadmap of the glycan utilization mechanisms deployed by individual members of the HGM is central to the development of dietary and microbial interventions to promote human health.

**FIG 1:**
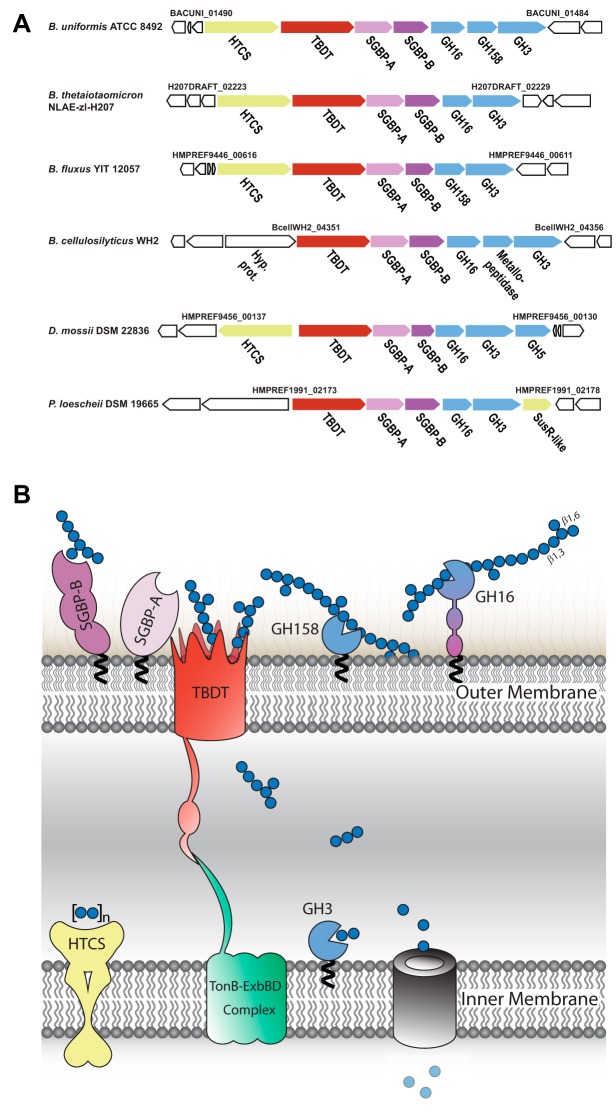
β(1,3)-Glucan utilization systems in the order *Bacteroidales*. (A) Strain-specific, syntenic β(1,3)-glucan utilization loci (1,3GUL) from Bacteroides uniformis, B. thetaiotaomicron, *B. fluxus*, *B. cellulosilyticus*, Dysgonomonas mossii, and Prevotella loescheii. Genome locus tags constituting 1,3GUL boundaries are indicated. Predicted or confirmed (this work) functional annotations are denoted below each gene. HTCS, hybrid two-component system sensor/regulator; SusR-like sensor-regulator; TBDT, SusC-like TonB-dependent transporter; SGBP-A, SusD-like cell-surface glycan-binding protein; SGBP-B, sequence-divergent cell-surface glycan-binding protein; GH*n*, member of glycoside hydrolase family *n*. (B) Model of B. uniformis laminarin utilization based on the present study and by analogy with the archetypal starch utilization system (Sus) (10, 13). Gene products are colored analogously to panel A, and predicted N-terminal lipidation following signal peptidase II cleavage is indicated with a black squiggle.

An explosion of (meta)genome sequence data continues to reveal substantial taxon-level variation in the metabolic potential of the HGM, yet a lack of functional data restricts our ability to fully explain or predict these differences and eventually to use this knowledge to engineer changes to the HGM. *Bacteroides* species, in particular, are predominant autochthonous members of the HGM that metabolize a wide variety of complex glycans into short-chain fatty acids (SCFAs) (11), which is enabled by a plethora of PULs in their genomes (12). As exemplified by the β(1,3)-glucan utilization loci (1,3GULs) elucidated here, PULs encode concerted molecular systems of surface glycan binding proteins (SGBPs), carbohydrate-active enzymes (CAZymes), TonB-dependent transporters (TBDTs), and sensor/regulators to recognize, capture, import, and saccharify individual substrates (13) (Fig. 1). Recently, a number of seminal, integrated PUL studies combining genetics, biochemistry, and structural biology have highlighted how strain-level genomic variation dictates nutrient specificities (14–27).

**FIG 2:**
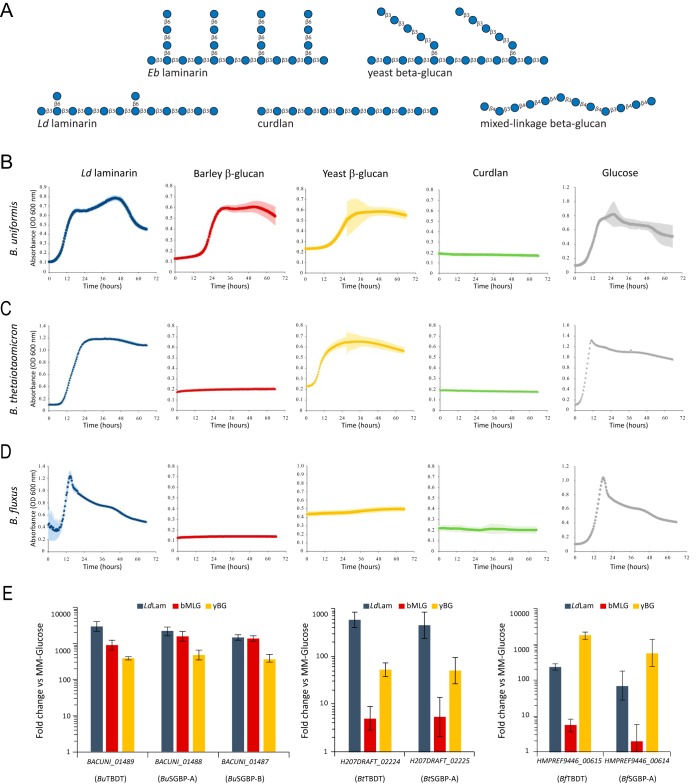
β(1,3)-Glucan utilization by *Bacteroides*. (A) Structures of β-glucans used in this study. Bacterial curdlan is a representative unbranched β(1,3)-glucan, analogous to plant callose. Algal laminarins are β(1,3)-glucans with β(1,6)-linked branches. *Laminaria digitata* laminarin has infrequent, single β(1,6)-glucosyl branches [β(1,3) to β(1,6) molar ratio, 7:1], while *Eisenia bicyclis* laminarin has a high frequency of branches with degrees of polymerization of up to three β(1,6)-glucosyl residues [β(1,3) to β(1,6) molar ratio, 3:2]. Yeast β(1,3)-glucan contains longer β(1,3)-glucan branches extended from β(1,6)-linked branch points. Cereal mixed-linkage β(1,3)/β(1,4)-glucans (MLGs) are linear chains of β(1,4)-linked cellotriosyl and cellotetraosyl units linked by β(1,3) bonds. (B to D) Growth curves of B. uniformis ATCC 8492 (B), B. thetaiotaomicron NLAE-zl-H207 (C), and *B. fluxus* YIT 12057 (D) in minimal medium containing the indicated carbon source at 0.5%, wt/vol. Points represent averages from *n *= 6 technical replicates (microplate wells) for polysaccharides and *n *= 3 for glucose; error bars represent the standard errors of the means. (E) RNA abundance for core PUL genes of *Bacteroides* spp. quantified by qRT-PCR. Bacteria were grown to mid-log phase in minimal medium containing glucose as the sole carbon source and subsequently exposed to different β-glucans (*n *= 3, expression measurements from individual cultures, relative to glucose control; error bars represent the standard errors of the means).

Amorphous β-glucans are ubiquitous polysaccharides in the human diet, which can be delineated broadly by backbone linkage (Fig. 2A): mixed-linkage β(1,3)/β(1,4)-glucans (MLGs) are commonly found in cereal crops such as oats and barley, while the β(1,3)-glucan callose is found as a component of plant cell walls (28). Edible fungi, including yeasts, contain β(1,6)-glucans (22) and β(1,3)-glucans (28). β(1,3)-Glucans also occur in the cell walls of seaweeds (29). Many β(1,3)-glucans also contain β(1,6)-linked branches, the length and frequency of which vary according to source (e.g., yeast β-glucan and laminarin) (28). It is known generally that a range of taxa in the HGM, including from the phyla *Firmicutes*, *Actinobacteria*, and *Bacteroidetes*, metabolize β-glucans to produce short-chain fatty acids (19, 22, 30–32). In addition to this nutritional benefit (6), β-glucans have been associated with health-promoting effects against cancer, diabetes/metabolic syndrome, and inflammation (33–36). However, the molecular mechanisms underpinning these effects have not been fully elucidated.

Previous studies on MLG and β(1,6)-glucan utilization by the symbiotic *Bacteroides* revealed the molecular details by which the different, dedicated PUL-encoded machineries target these two distinct classes of β-glucans (19, 22, 26). Here, we functionally dissected an exemplar 1,3GUL from Bacteroides uniformis ATCC 8492 to provide molecular insight into β(1,3)-glucan utilization, thereby resolving a key outstanding deficit in our understanding of β-glucan metabolism by the HGM. Notably, this included solving the first tertiary structure and resolving the catalytic mechanism of a member of the new glycoside hydrolase family 158 (GH158). Building upon these foundational results, we subsequently demonstrated that the individual abilities of three *Bacteroides* species to metabolize distinct β(1,3)-glucans and/or MLG is dictated by the cumulative specificities and contributions of their respective SGBPs, cell surface GHs, and other sensor/transport functions encoded by partially homologous 1,3GULs. Finally, we found, through metagenomic analysis, that the prevalence of 1,3GULs in human gut microbiomes is species-dependent but broadly distributed worldwide.

## RESULTS

### B. uniformis possesses a distinct PUL upregulated during growth on β(1,3)-glucans.

B. uniformis ATCC 8492 exhibited robust growth in minimal medium containing either glucose or the branched β(1,3)-glucans from Laminaria digitata [*L. digitata* laminarin, or *Ld*Lam, which contains single β(1,6)-linked glucosyl branches] and Saccharomyces cerevisiae [yeast β-glucan, yBG, which contains longer β(1,6)-linked glucosyl branches extended by β(1,3)-linked glucose units]. No growth was observed on Alcaligenes faecalis curdlan [an unbranched β(1,3)-glucan analogous to plant callose], which is poorly soluble and forms a gel in water (Fig. 2B and Table S1 at https://doi.org/10.14288/1.0388792). Interestingly, B. uniformis also grew well on barley MLG [bMLG, which has an ∼2.5:1 ratio of β(1,4) to β(1,3) backbone linkages] (Fig. 2B and Table S1 at https://doi.org/10.14288/1.0388792) despite lacking a canonical mixed-linkage glucan utilization locus (MLGUL) homologous to the Bacteroides ovatus MLGUL (19).

We identified in B. uniformis a putative β(1,3)-glucan utilization locus that encodes a TonB-dependent transporter (TBDT; a SusC homolog, BACUNI_01489) and a cell surface glycan-binding protein (SGBP-A; a SusD homolog, BACUNI_01488) as canonical PUL signatures (37), as well as an additional nonhomologous SGBP (*Bu*SGBP-B; BACUNI_01487), three glycoside hydrolases (GH16 subfamily 3, GH158, and GH3; BACUNI_01486 to _01484), and a hybrid two-component system (HTCS) transcriptional regulator (BACUNI_01490) (Fig. 1). In particular, the GH complement was suggestive of a role in β(1,3)-glucan hydrolysis: GH16 subfamily 3 (GH16_3 [38]) contains known *endo*-laminarinases (among other *endo*-β-glucanases) (19) and GH3 contains *exo*-β-glucosidases (among others) (39). Notably, during the course of this study, GH158 emerged as a new family whose founding member was shown to hydrolyze a chemically derivatized β(1,3)-glucan in a high-throughput screen (40). Concordant with this proposed PUL specificity, when we probed the expression of core genes encoding the TBDT and both SGBPs (BACUNI_01487-01489), we found that they were strongly upregulated in the presence of laminarin, bMLG, and yBG as sole carbon sources versus a glucose control (Fig. 2E). The syntenic TBDT and SGBP-A (SusC/SusD homologs) in a partially homologous Bacteroides cellulosilyticus WH2 PUL (Fig. 1) were shown previously to be similarly upregulated in the presence of laminarin and bMLG (41). Notably, *B. ovatus* ATCC 8483, which possesses an MLGUL (19) but not a homologous 1,3GUL (Fig. 1), does not grow on laminarin (37).

### Biochemical basis of β(1,3)-glucan recognition and degradation by B. uniformis.

By analogy with other PUL-encoded systems, we propose a working model of concerted β(1,3)-glucan saccharification and uptake by the proteins of the 1,3GUL (Fig. 1). This model involves polysaccharide capture at the cell surface by at least one SGBP, backbone hydrolysis by at least one *endo*-glucanase, transport of oligosaccharide fragments through the outer membrane by the TBDT, and ultimate saccharification by an *exo*-β-glucosidase in the periplasm. Indeed, signal peptide analysis with SignalP, LipoP, and PSORTb predicted that *Bu*GH16 is localized at the cell surface via N-terminal Cys lipidation (PSORTb score, 9.7), whereas *Bu*GH3, which also harbors a type II signal peptide, is predicted to be periplasmic (PSORTb score, 9.4). Interestingly, PSORTb was unable to predict the localization of *Bu*SGBP-A, *Bu*SGBP-B, and *Bu*GH158 (score, <2.5) despite the presence of a type II signal peptide, a +2 serine residue in each, and expected extracellular localization of at least the SGBPs by analogy with the archetypal starch utilization system (9, 10).

**(i) *Bu*GH16 is a broad-specificity *endo-*β(1,3)/β(1,4)-glucanase.** To investigate the catalytic role of *Bu*GH16 in surface polysaccharide breakdown, the hydrolytic activity of the recombinant protein was screened against a library of polysaccharides (Table S2 at https://doi.org/10.14288/1.0388792). *Bu*GH16 displayed activity toward the β(1,3)-glucans *Ld*Lam, Eisenia bicyclis laminarin [*Eb*Lam, which contains more frequent β(1,6)-linked glucosyl branches of up to three β(1,6)-linked glucosyl residues (42); Fig. 2A], yBG, and curdlan. *Bu*GH16 was also active on bMLG. Using laminarin as the substrate, the pH optimum was 6.0 (consistent with extracellular function in the human large intestine [43]), and the maximum temperature of activity was ca. 50°C (Fig. S1 at the URL mentioned above).

**FIG 3:**
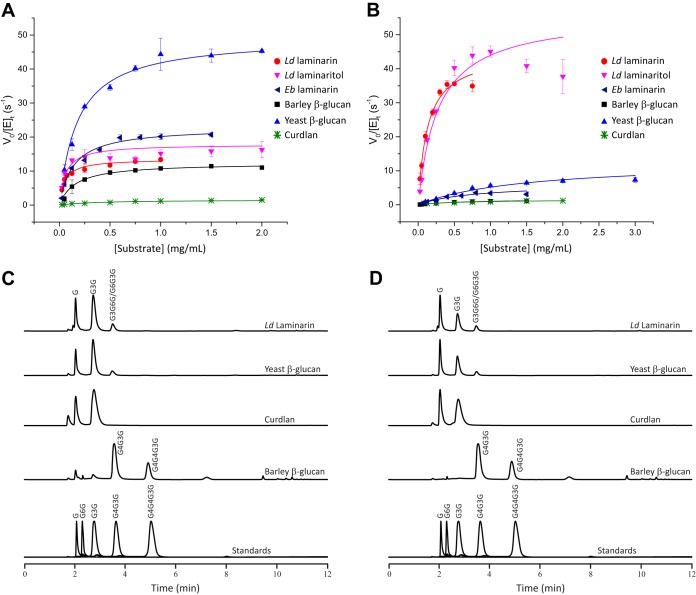
Hydrolysis of β-glucans by *Bu*GH16 and *Bu*GH158. (A and B) Initial-rate kinetics analysis of *Bu*GH16 (A) and *Bu*GH158 (B). *V*_0_/[*E*]_*t*_ = initial rate per total enzyme concentration. Curves represent fits of the Michaelis-Menten equation to the average data points (*n *= 3); error bars represent standard deviations from the means. (C and D) HPAEC-PAD analysis of limit-digest products of *Bu*GH16 (C) and *Bu*GH158 (D) at 37°C.

Subsequent Michaelis-Menten kinetics at the optimum pH and 37°C confirmed that *Bu*GH16 is a broadly specific β(1,3)-glucanase with similar catalytic efficiencies toward laminarins and yBG (Fig. 3A and Table S2 at the URL mentioned above). The *k*_cat_/*K*_m_ value of *Bu*GH16 on bMLG was 4-fold lower than those for these all-β(1,3)-backbone glucans (Fig. 3A and Table S2 at the URL mentioned above). The enzyme was very poorly active on the unbranched β(1,3)-glucan, curdlan (*k*_cat_/*K*_m_ value ca. 1% of that on the laminarins) (Fig. 3A and Table S2 at the URL mentioned above). *Ld*Lam and yBG ultimately were hydrolyzed to glucose, laminaribiose, and a trisaccharide (G3G6G or G6G3G), as determined by high-performance anion-exchange chromatography with pulsed amperometric detection (HPAEC-PAD) (Fig. 3C) and confirmed by matrix-assisted laser desorption ionization mass spectrometry of the per-*O*-acetylated crude mixture (data not shown). The limit-digest products were glucose and laminaribiose from curdlan, and G4G3G and G4G4G3G from bMLG (Fig. 3C). Analyses of the hydrolysis products over time suggest that *Bu*GH16 operates by an *endo*-dissociative mode of action (Fig. S2 at the URL mentioned above).

Despite extensive attempts using the full-length protein and the GH16 module only (Fig. S3 at the URL mentioned above), we were not able to obtain a crystal structure of *Bu*GH16 to explain the observed substrate promiscuity. However, protein phylogeny unambiguously places *Bu*GH16 in the “β-bulge laminarinase/MLGase” clade (Fig. S4 at https://doi.org/10.14288/1.0388792), corresponding to GH16 subfamily 3 (38), which is known to contain laminarinases with secondary MLGase activity, as well as bona fide MLGases (19). These enzymes require a β(1,3) linkage between the −1 and −2 subsites (44), but beyond the −2 subsite, requirements are less stringent, leading to widespread promiscuity in polysaccharide and bond cleavage specificity [β(1,3) versus β(1,4)] (19). Notably, structural homology modeling and superposition with an exemplar laminarinase from the marine bacterium Zobellia galactanivorans DsijT (45) rationalizes the ability of *Bu*GH16 to accommodate highly branched β(1,3)-glucans, i.e., laminarins and yBG (Fig. S5 at the URL mentioned above).

In addition to the catalytic module, *Bu*GH16 comprises a pair of PFAM 13004 domains (Fig. S3 at the URL mentioned above). Despite initial bioinformatics predictions, carbohydrate binding has not been demonstrated for any PFAM 13004 domain to date (15). Likewise, affinity gel electrophoresis (AGE) analysis of a recombinant protein consisting of the two PFAM 13004 domains of *Bu*GH16 revealed that they do not bind cognate polysaccharides of the 1,3GUL (Fig. S6 at the URL mentioned above). These domains likely serve a spacer function, as in *B. ovatus* GH5 (*Bo*GH5) (15), and are analogous to all β-sheet domains in SGBPs (16, 26, 46).

**(ii) *Bu*GH158 is a strictly specific, retaining *endo*-β(1,3)glucanase with a triose phosphate isomerase (TIM) barrel fold.** *Bu*GH158 is a member of a newly established GH family, the distantly related founding member of which, Vvad_PD1638 (sequence identity 29%) (Fig. S7 at https://doi.org/10.14288/1.0388792), was shown to be active on the artificial proxy substrate, carboxymethyl-curdlan, in a high-throughput screen (40). Hence, we performed detailed kinetic and product analysis to more precisely delineate the specificity of *Bu*GH158 in the context of the 1,3GUL (Fig. 3B and Table S2 at the URL mentioned above). In contrast to *Bu*GH16, *Bu*GH158 is highly specific for *Ld*Lam, with a *k*_cat_/*K*_m_ value ca. 2 orders of magnitude higher than those for *Eb*Lam, yBG, MLG, and curdlan (Fig. 3B; also see and Table S2 and Fig. S1 and S2 at the URL mentioned above). The corresponding hydrolysis products were identical to those of *Bu*GH16 (Fig. 3D).

**FIG 4:**
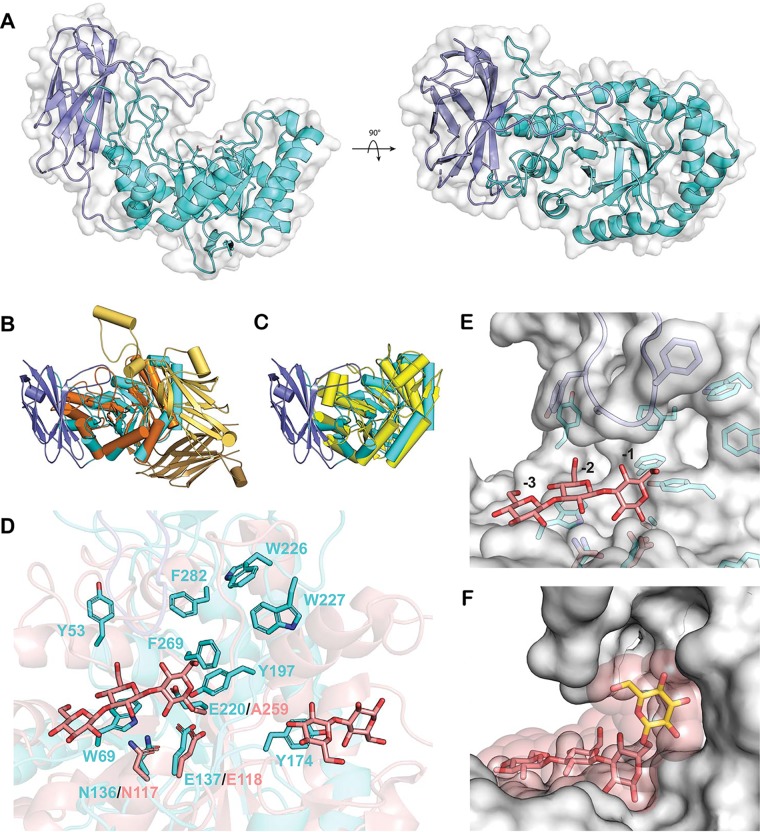
*Bu*GH158 tertiary structure. (A) Overall structure of *Bu*GH158, with the TIM barrel domain colored cyan, the Ig-like domain colored slate, and a semitransparent surface in white. The catalytic residues E137 and E220 are shown as sticks. (B) Superposition of *Bu*GH158 (colored as described for panel A) with the Bifidobacterium dentium GH2 β-glucuronidase, which has two additional domains N terminal to the TIM barrel (PDB entry 5Z1B, orange). (C) Superposition of *Bu*GH158 (colored as described for panel A) and the *Chrysonilia sitophila* GH5 β(1,4)-mannanase (PBD entry 4AWE, yellow), which has no additional domains. (D) Superposition of *Bu*GH158 (colored as described for panel A) with a laminaritriose complex of a GH17 *endo*-β(1,3)-glucanase from Solanum tuberosum (*St*GH17; PDB entry 4GZJ, rose). *Bu*GH158 aromatic residues in the active-site cleft are shown in cyan sticks, and homologous catalytic residues in *St*GH17 are shown in rose sticks. (E) Surface representation of *Bu*GH158 superposed with the *St*GH17 laminaritriose ligand (rose sticks) occupying the negative subsites as labeled, revealing a pocket near 6-OH of the glucose in the −1 subsite. (F) A glucose molecule, shown as yellow sticks, attached to the subsite −1 glucose via a β(1,6)-linkage. The transparent sphere about each atom of the modeled glucose and laminaritriose represent a van der Waals radius of 1.5 Å.

To provide the first three-dimensional insights into substrate specificity and catalysis in GH158, we determined the tertiary structure of the enzyme to 1.8 Å by X-ray crystallography (Table S3 at the URL mentioned above). *Bu*GH158 consists of an N-terminal (α/β)_8_ TIM barrel domain and a C-terminal, eight-stranded immunoglobulin (Ig)-like domain in contact with helices α7 and α8 of the TIM barrel. Additional loops contribute to the extensive contact between domains, with one from the Ig-like domain extending above the TIM barrel to shape the active-site cleft (Fig. 4A). A Dali structure similarity search returned members of clan GH-A as the top 20 structural homologs (Table S4 at the URL mentioned above), confirming the bioinformatics prediction that GH158 constitutes an evolutionarily distinct family within this clan (40). Correspondingly, superposition with the top two GH2 and GH5 results show that the TIM barrel core is well conserved and that family-level differences arise from the presence or absence of accessory domains (Fig. 4B and C).

All clan GH-A enzymes are predicted to use a canonical Koshland double-displacement mechanism employing a covalent glycosyl enzyme intermediate and resulting in overall retention of the anomeric stereochemistry at the site of polysaccharide backbone hydrolysis (39). This was confirmed for *Bu*GH158 by nuclear magnetic resonance (NMR) analysis of the hydrolysis of 2-chloro-4-nitrophenyl laminaribioside (G3G-CNP), which constitutes the first stereochemical determination for the family (Fig. S8 at the URL mentioned above). Clan GH-A members present the conserved catalytic acid/base and nucleophile residues on loops immediately following strands β4 and β7, corresponding to E137 and E220, respectively, in *Bu*GH158 (Fig. 4D). Additionally, a conserved asparagine (N136) precedes the general acid/base (E137) and is anticipated to engage in hydrogen-bonding interactions with the substrate (Fig. 4D) (39). Indeed, site-directed mutation of N136, E137, or E220 to alanine completely abolished catalytic activity (data not shown).

GH158 is the fourth clan GH-A family, in addition to GH17, -128, and -148, now known to contain an *endo*-β(1,3)-glucanase activity (39, 40). The active-site cleft surrounding the catalytic sidechains is rich in surface-exposed aromatic residues oriented to engage in stacking interactions with the substrate (Fig. 4D). Structural alignment with a Solanum tuberosum GH17 *endo*-β(1,3)-glucanase:laminaritrose (G3G3G) complex (47) (root mean square deviations, 3.1 Å; sequence identity, 20.6%) infers the directionality of polysaccharide binding in *Bu*GH158 (Fig. 4D). Crucially, this superposition also reveals that 6-OH of the glucosyl residue in the −1 subsite is oriented toward a pocket lined with aromatic residues that may accommodate a single β(1,6)-linked glucose branch in *Bu*GH158 (Fig. 4E). Indeed, a glucosyl residue could easily be manually docked into this position without clashes (Fig. 4F). This structural feature provides a plausible explanation for the strict *Ld*Lam specificity and poor activity on yBG, the latter of which contains longer β(1,6)-linked glucose branches (48) (Fig. 2A).

**(iii) Periplasmic saccharification of imported oligosaccharides is mediated by *Bu*GH3, a specific *exo*-β(1,3)-glucosidase.** GH3 is known to contain members with a diversity of *exo*-β-glycosidases (39), warranting detailed kinetic characterization of *Bu*GH3 in the context of the 1,3GUL. Initial substrate screening on chromogenic *p*NP glycosides revealed that *Bu*GH3 is an *exo-*β-glucosidase (no other activity detected on a panel of *p*NP substrates; see Table S5 at https://doi.org/10.14288/1.0388792). Subsequent Michaelis-Menten kinetics on diverse β-gluco-oligosaccharides further established that *Bu*GH3 is a specific β(1,3)-glucosidase poised to efficiently handle the hydrolysis products of the predicted cell surface GH16 and GH158 enzymes (Table S5 and Fig. S9 at the URL mentioned above). Indeed, incubation with the limit-digest products of either *Bu*GH16 or *Bu*GH158 confirmed that *Bu*GH3 is capable of completely degrading all oligosaccharide products of laminarin and bMLG to glucose (Fig. S2 and S10 at the URL mentioned above).

Despite possessing an apparently broad ability to hydrolyze β(1,3)-, β(1,4)-, and β(1,6)-glucosides, the particular preference of *Bu*GH3 for β(1,3)-linkages is highlighted by a two-orders-of-magnitude higher *k*_cat_/*K*_m_ value for laminaribiose (G3G) over cellobiose (G4G) and gentiobiose (G6G) (Table S5 at the URL mentioned above). Concordant with this finding, catalytic efficiency toward mixed-linkage trisaccharides with a β(1,3)-glucose at the nonreducing end (G3G4G) was 2 orders of magnitude higher than that with a β(1,4)-glucose at the nonreducing end (G4G3G) (Table S5 at the URL mentioned above). Laminari-oligosaccharides of increasing degree-of-polymerization were hydrolyzed with comparable *k*_cat_ values, but *K*_m_ values decreased between the di- and trisaccharide, after which *K*_m_ values leveled off, suggesting that the *Bu*GH3 has two positive subsites of kinetic significance.

**FIG 5:**
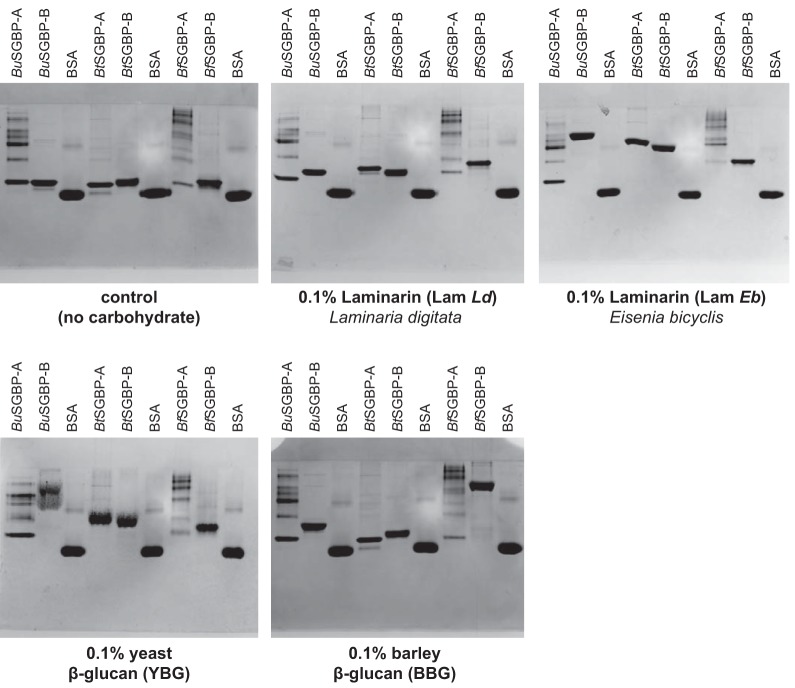
Binding of *Bacteroides* SGBPs to β(1,3)-glucans. Native polyacrylamide (10%) gel electrophoresis containing 0.1% polysaccharide, with bovine serum albumin (BSA) as a control protein.

**(iv) *Bu*SGBP-B mediates β-glucan specificity.** The 1,3GUL encodes two potential SGBPs: the SusD-homolog *Bu*SGBP-A and the sequence-divergent, “SusE-positioned” (13) *Bu*SGBP-B. Notably, qualitative screening of a library of soluble polysaccharides by affinity gel electrophoresis (AGE), as well as isothermal titration calorimetry (ITC), indicated that *Bu*SGBP-A does not bind any likely substrates, including *Ld*Lam and bMLG (Fig. 5; see also Tables S6 and S7 and Fig. S11 and S12 at https://doi.org/10.14288/1.0388792). SGBP-A homologs that do not bind polysaccharide currently are rare but not entirely unknown (25); indeed, the primary role of this *Bacteroides* PUL component appears to be its indispensable structural association with the cognate TBDT (16, 26, 46, 49).

In contrast, AGE demonstrated that *Bu*SGBP-B was able to bind *Ld*Lam, *Eb*Lam, yBG, and bMLG (Fig. 5). The smaller shift observed for *Ld*Lam than for *Eb*Lam (Fig. 5) recapitulates observations for an SGBP-A from a marine *Bacteroidetes*, *Gramella* sp. (50). Quantitative ITC indicated an order-of-magnitude-higher affinity constant (*K*_a_) for *Ld*Lam over bMLG (Table S6 at the URL mentioned above), revealing the high specificity of *Bu*SGBP-B for β(1,3)-glucan. The smallest laminari-oligosaccharide bound by *Bu*SGBP-B was the trisaccharide (G3G3G), and affinity increased with degree of polymerization (Table S7 and Fig. S12 at the URL mentioned above). Neither SGBP recognized insoluble crystalline cellulose [β(1,4)-glucan] in pulldown assays (data not shown), unlike SGBPs that bind MLG (26), which further underscores the β(1,3)-glucan specificity of *Bu*SGBP-B.

### Divergent GH and SGBP specificities collectively dictate the range of β-glucans utilized by *Bacteroides* species.

Using the B. uniformis 1,3GUL as the archetype, we identified several homologous 1,3GULs from the *Bacteroidaceae*, *Porphyromonadaceae*, and *Prevotellaceae* families (order *Bacteroidales*), of which five representatives are shown in Fig. 1. All comprise a syntenic TBDT, SGBP-A, SGBP-B, and GH3 as the core set of conserved genes. However, the predicted *endo*-glucanase (GH16 and GH158) content is notably variable, and the SGBPs have particularly low sequence similarity, including among related *Bacteroides* species (Fig. 1 and Table S8 at https://doi.org/10.14288/1.0388792). With a focus on this genus, we tested the growth of B. thetaiotaomicron and B. fluxus *vis-à-vis* B. uniformis to determine how heterogeneity in 1,3GUL gene content might affect the utilization of individual β-glucans. Strikingly, whereas all three species were able to grow on *Ld*Lam as the sole carbon source, only B. uniformis and B. thetaiotaomicron grew on yBG, while only B. uniformis grew on bMLG (Fig. 2B to D and Table S1 at the URL mentioned above).

To elucidate the molecular basis of this species-specific β-glucan utilization, we characterized the transcriptional response of the B. thetaiotaomicron and B. fluxus 1,3GUL (as a measure of HTCS specificity) and the biochemistry of the predicted surface *endo*-glucanases and SGBPs *vis-à-vis* the B. uniformis system. In B. thetaiotaomicron and B. fluxus, the genes encoding the TBDT and SGBP-A were strongly upregulated by *Ld*Lam and yBG, whereas they were only very weakly activated by bMLG (<10-fold) (Fig. 2E). These results are generally concordant with the observed growth phenotypes among all three *Bacteroides* species, although we unexpectedly observed strong upregulation of the B. fluxus 1,3GUL (*Bf*1,3GUL) with yBG, on which B. fluxus is not able to grow. The *Bf*1,3GUL still was highly activated after dialyzing the substrate to remove any potential small oligosaccharide signals (Fig. S13 at the URL mentioned above), suggesting that this system is capable of sufficiently cleaving yBG to release an oligosaccharide that activates this PUL but subsequently unable to grow appreciably on the bulk polysaccharide. These distinct regulatory profiles likely involve a combination of surface GH and transporter specificity, combined with the sensing abilities of the respective HTCS regulators. The latter signaling specificity is notable in light of high protein sequence similarity among species (80 to 85% amino acid identity and 88 to 91% similarity versus the *Bu*HTCS; Table S8 at the URL mentioned above), and the results below show that at least B. thetaiotaomicron GH16 (*Bt*GH16) can cleave bMLG but does generate an activating cue.

Whereas B. uniformis encodes both a GH16_3 member and a GH158 member in its 1,3GUL, B. thetaiotaomicron possesses only a GH16_3 member while B. fluxus possesses only a GH158 member. Enzymology revealed that *Bt*GH16 is a predominant laminarinase with lower, yet comparable, activity on yBG and bMLG, similar to *Bu*GH16 (Fig. S14 and Table S2 at the URL mentioned above; the homologous Prevotella loescheii GH16 [*Pl*GH16] also had broad activity). In contrast, *Bf*GH158 was highly specific for *Ld*Lam, with low activity on yBG and very poor activity on bMLG, similar to *Bu*GH158 (Table S2 at the URL mentioned above).

We also assessed the β-glucan specificities of the SGBPs from the B. thetaiotaomicron and B. fluxus 1,3GULs for comparison with those of B. uniformis. *Bt*SGBP-A, *Bt*SGBP-B, *Bf*SGBP-A, and *Bf*SGBP-B each bound the all-β(1,3)-linked *Ld*Lam, *Eb*Lam, and yBG, as shown by AGE and ITC, although *Bf*SGBP-A and -B interacted only weakly with yBG (Fig. 5 and Table S6 and Fig. S11 at the URL mentioned above). Strikingly, *Bf*SGBP-B migration was strongly hindered by the β(1,3)/β(1,4)-linked bMLG in AGE, whereas that of *Bf*SGBP-A, *Bt*SGBP-A, and *Bt*SGBP-B was unaffected. Indeed, ITC indicated that *Bf*SGBP-B bound bMLG with an affinity similar to that of *Bu*SGBP-B (Table S6 at the URL mentioned above).

**FIG 6:**
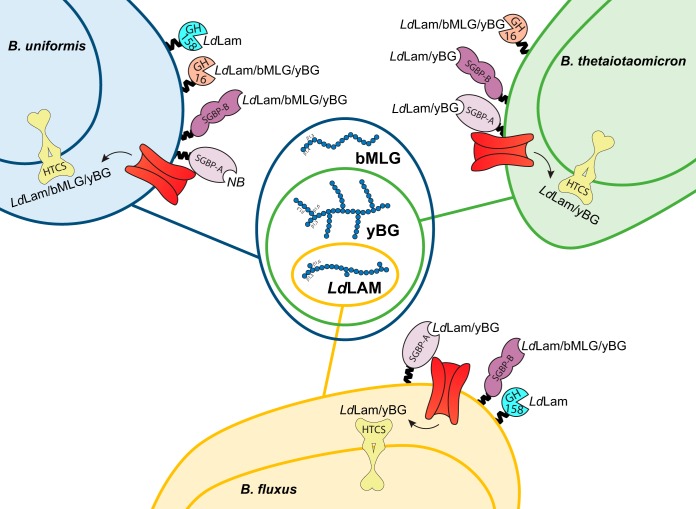
Diagram of β-glucan utilization by *Bacteroides* of the HGM. For each species, the binding or catalytic specificity of the proteins encoded by the endogenous 1,3GUL is indicated on the cell surface (Lam, laminarin; bMLG, barley mixed-linkage β-glucan; yBG, yeast β-glucan; NB, no binding). The capacity of each species to utilize individual β-glucans for growth, which arises as a combination of these individual specificities, as well as periplasmic transport/sensing, is indicated in the Venn diagram.

These biochemical data show that whereas orthologous GH specificity is well predicted by the family to which it belongs, considerable diversity exists in the specificity of syntenic SGBPs encoded by 1,3GULs of B. uniformis, B. thetaiotaomicron, and B. fluxus. When combined with microbiological data, a pattern emerges whereby GH content combined with SGBP specificity predicts the range of β-glucan congeners a *Bacteroides* species can utilize. In parallel, the signaling specificity or promiscuity through the single HTCS sensor associated with each system must also be able to respond to the diversity of cues generated from cleavage of related substrates. Of the three species examined, only B. uniformis possesses the full complement of synergistic HTCS, GH, and SGBP specificities to enable growth on laminarin, yBG, and bMLG (summarized in Fig. 6).

### Metagenomic survey reveals global 1,3GUL distribution in the human gut.

**FIG 7:**
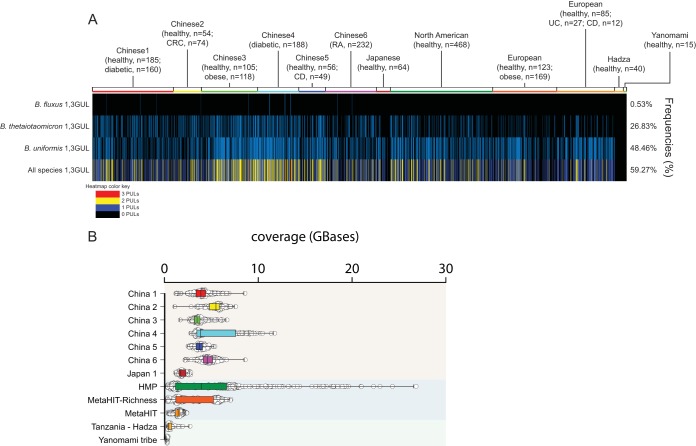
Prevalence of 1,3-GULs in 2,441 human metagenomes. (A) Each line represents the presence (blue) or absence (black) of a specific 1,3GUL species related to a single human gut metagenomic sample. The bottom row represents the total number of 1,3GUL that each individual possesses, colored according to the legend. The frequency of 1,3GUL incidence across all 2,441 individuals is shown on the right. (B) Coverage variation of each metagenomic sample is indicated by individual samples (open circles) and a box plot with the mean.

To determine the prevalence of 1,3GULs in the human gut, we surveyed publicly available gut metagenomic data from 2,441 adults across five different continents (North American, South America, Africa, Europe, and Asia) (Fig. 7). Despite similar genetic synteny, these 1,3GULs have different nucleotide sequences (pairwise identities for B. fluxus/B. thetaiotaomicron, 44%; B. fluxus/B. uniformis, 60%; B. thetaiotaomicron/B. uniformis, 44%), allowing us to use the nucleotide sequence to distinguish the presence or absence of a specific 1,3GUL in each metagenomic sample. The B. uniformis type 1,3GUL is the most abundant across all samples (48% of the total population), perhaps reflecting the high prevalence of this species in humans from industrialized populations. This is followed by the B. thetaiotaomicron type 1,3GUL (26% of samples) and *B. fluxus* (0.53% of samples). The low prevalence of *B. fluxus* may be explained by its low abundance and prevalence in European, North American, and Asian populations, consistent with previous observations for the xyloglucan utilization locus (15). Although 1,3GULs are widely distributed throughout human populations (59%), we do not see correlation with any particular geographic region or population. This ubiquity may be explained by the prevalence of β(1,3)-glucans in different diets, since edible fungi and yeast fermentation products are common worldwide. Strikingly, we were unable to detect any of the three identified 1,3GULs in the indigenous Hadza and Yanomami tribes, which may be due to a high prevalence of *Prevotella* and not *Bacteroides* in these populations (51, 52). The present study, in combination with our previous metagenomic survey of MLG utilization (19), reveals a broadly represented potential for specific β-glucan metabolism to establish niches for individual human gut bacteria.

## DISCUSSION

The human large intestine is a highly competitive ecosystem, in which access to a wide range of carbohydrates confers selective advantage. Here, we outline a model in which a single PUL enables B. uniformis to utilize a range of β-glucan congeners (Fig. 1). In this model, *Ld*Lam, yBG, or MLG (Fig. 2) is bound by *Bu*SGBP-B on the surface of the bacterium, whereas the SusD homolog *Bu*SGBP-A is passive in this step. Depending on the backbone linkage composition and extent of branching, individual β-glucans are cleaved into oligosaccharides by one or both cell surface-anchored *endo*-glucanases. Whereas *Bu*GH158 is a highly specific laminarinase, *Bu*GH16 is a generalist *endo*-β(1,3)-glucanase that can accommodate backbone β(1,4)-glucosyl linkages (as in MLG) and long/frequent β(1,6)-glucosyl branches (as in *Eb*Lam and yBG). Oligosaccharides produced by these *endo*-glucanases are actively imported by the associated TBDT into the periplasm, where the *exo*-β-glucosidase *Bu*GH3 completes saccharification. Notably, the poor activity of *Bu*GH3 toward β(1,6) and β(1,4) linkages suggests that other periplasmic glucosidases encoded outside 1,3GUL assist with oligosaccharide debranching and complete MLG oligosaccharide hydrolysis. The genome of B. uniformis ATCC 8492 encodes three members of GH30, which is known to contain *exo-*β(1,6)-glucosidases active on laminarin (53), as well as twenty additional members of GH3, which could comprise a potent *exo-*β(1,4)-glucosidase.

The *Bacteroides* 1,3GULs characterized here share partial homology with various laminarin-targeting PULs from marine *Bacteroidetes*, including *Gramella* (54), *Formosa* (55), and other closely related genera within the family *Flavobacteriaceae* (56), raising questions of evolutionary origin of 1,3GULs in the HGM. Indeed, PULs that target porphyran (a galactan-based seaweed polysaccharide) are thought to have been acquired by the HGM from the marine *Bacteroidetes* Zobellia galactanivorans via a horizontal gene transfer event (57). In addition to syntenic 1,3GULs, recently characterized *Formosa* species also harbor enlarged laminarin-targeting PULs containing other enzymes. In this system, laminarin must first be debranched by a GH30 *exo*-β(1,6)-glucosidase before being hydrolyzed by a GH17 *endo*-β(1,3)-glucanase, which has very low activity on β(1,6)-glucose-branched laminarin (55). In general, GH17 endoglucanases have a very narrow active-site cleft that does not effectively accommodate such branches (47, 55). This is unlike *Bu*GH16 and *Bu*GH158 enzymes, which allow the 1,3GUL system to bypass the initial debranching requirement. Thus, although GH16 and GH17 laminarin-active enzymes may display similar overall β(1,3) linkage specificities (58), differences in active-site structure may delineate distinct roles in the stepwise total hydrolysis of laminarins.

Comparative genomic and biochemical analysis revealed variations in GH content that, together with complementary HTCS, GH, and SGBP specificity, dictate selective β-glucan utilization. Among the *Bacteroides* species tested, B. uniformis is unique in its ability to utilize the trifecta of β-glucans containing contiguous or individual β(1,3) linkages, *viz.*, *Ld*Lam, yBG, and bMLG. In contrast, B. thetaiotaomicron can only access *Ld*Lam and yBG, while B. fluxus is further restricted to utilizing *Ld*Lam only (summarized in Fig. 6). None of the species tested were able to utilize curdlan, perhaps due to its poor solubility in water. Orthologous GHs from syntenic 1,3GUL share similar specificities, yet the GH complement alone is insufficient to confer growth on a particular β-glucan. For example, B. thetaiotaomicron produces a GH16 member that is an approximately equally competent laminarinase, yBGase, and MLGase (see Table S2 at https://doi.org/10.14288/1.0388792). However, an inability to capture bMLG at the cell surface, due to a lack of polysaccharide binding by the cognate SGBPs (Fig. 5), and an inability to transport or recognize breakdown products in the periplasm (Fig. 2) precludes growth on this abundant dietary glycan. It is interesting to ponder to what extent losses or gains of function are correlated and arise (a)synchronously through the evolution of these distinct protein families more widely among the *Bacteroidetes*.

In this regard, B. fluxus is the least versatile of the three *Bacteroides* species explored here. The inability of B. fluxus to utilize bMLG clearly is the consequence of three factors (Fig. 6): (i) the lack of a polyspecific GH16 ortholog encoded by its 1,3GUL (cf. B. uniformis and B. thetaiotaomicron), (ii) the extremely poor MLGase activity of *Bf*GH158 (Table S2 at the URL mentioned above), and (iii) an inability to transport/sense this cereal polysaccharide in the periplasm (Fig. 2). However, we note that B. fluxus possesses an SGBP-B able to bind both laminarin and bMLG (Fig. 5 and Table S6 at the URL mentioned above), which is correlated with MLG utilization in the absence of an MLG-binding SGBP-A in B. uniformis (Fig. 6). Further, the inability of B. fluxus to grow on yBG is consistent with weak binding by *Bf*SGBP-A and *Bf*SGBP-B, poor catalytic efficiency of *Bf*GH158 toward yBG, and the lack of a compensatory GH16 ortholog. This is despite an HTCS that is equally highly responsive to yBG and *Ld*Lam (Fig. 2), as well as both SGBP types binding both of these 1,3-β-glucans. Hence, we might anticipate that a gain-of-function mutation to introduce yBG hydrolase activity, for example, through acquisition of a versatile GH16 member or broadening the substrate scope of the extant GH158, would result in growth on yBG. Testing this hypothesis will depend on the future generation of a transformation system for B. fluxus or discovery of a corresponding wild-type strain.

The present study underscores that dietary specificity of related gut commensal strains is gleaned most precisely by systems-based approaches involving genomic, biochemical, and structural biological dissection of PULs (14–27), which otherwise could not have been predicted based on sequencing data alone. Whereas the GH complement of PULs has been shown to drive specificity in the levan/fructan system (14), to our knowledge the present study describes the first case in which the interplay of GHs and SGBPs, underpinned by HTCS specificity, collectively dictate glycan utilization among species. Indeed, in most PULs characterized to date, the specificities of the vanguard GH and SGBPs are concordant (15, 17, 19, 23, 25, 26, 46). On the other hand, the B. fluxus case reveals how a limited endoglycanase in the context of polyspecific SGBPs and an HTCS can restrict nutrient range. Thus, the evolution of both the gene complement and the specificity of individual components within a PUL allows bacteria to access to distinct nutrient niches in the competitive human gut environment. In the context of human nutrition and health, the detailed characterization presented here provides a validated set of molecular markers (Fig. 1) to identify β(1,3)-glucan utilization potential among members of the HGM (and other microbiota). This insight may prove especially transformative for our future ability to select strains and dietary formulations in tailoring microbial intervention therapies (59).

## MATERIALS AND METHODS

### Substrates.

Tamarind seed xyloglucan, barley beta-glucan (60), konjac glucomannan, carob galactomannan, Alcaligenes faecalis curdlan, and yeast beta-glucan (48) were purchased from Megazyme International (Bray, Ireland). Carboxymethyl cellulose was purchased from Acros Organics (Morris Plains, NJ, USA). Hydroxyethyl cellulose was purchased from Amresco (Solon, OH, USA). Xanthomonas campestris xanthan gum was purchased from Spectrum (New Brunswick, NJ, USA). Laminarins from Laminaria digitate (61) and Eisenia bicyclis (42) were purchased from Sigma-Aldrich (St. Louis, MO, USA) and Carbosynth (Compton, UK), respectively. Laminarin from *Laminaria digitata* was reduced to laminaritol, as described previously (62), to reduce background responses in the bicinchoninic acid (BCA) reducing-sugar enzyme kinetics assay.

Laminaribiose (G3G), laminaritriose (G3G3G), laminaritetraose (G3G3G3G), laminaripentaose (G3G3G3G3G), mixed-linkage glucotriose A (G3G4G), mixed-linkage glucotriose B (G4G3G), and cellotriose (G4G4G) were purchased from Megazyme. Gentiobiose (G6G) was purchased from Carbosynth (Compton, UK). Cellobiose (G4G) was purchased from Acros Organics. G3G-CNP was synthesized by glycosylation of α-laminaribiosyl bromide (63) with 2-chloro-4-nitrophenol under phase-transfer conditions (63, 64), the details of which will be published elsewhere.

### Bacterial growth experiments.

Bacteroides uniformis ATCC 8492 (NCBI accession no. NZ_AAYH00000000.2, JGI accession no. 641380447), Bacteroides thetaiotaomicron NLAE-zl-H207 (NCBI accession no. NC_004663.1, JGI accession no. 2515154063), and Bacteroides fluxus YIT12057 (NCBI accession no. NZ_AFBN00000000.1, JGI accession no. 651324011) were grown in tryptone yeast extract glucose (TYG) medium at 37°C under anaerobic conditions (Coy anaerobic chamber; 85% N_2_, 10% H_2_, 5% CO_2_). These cultures were used to inoculate minimal medium containing glucose as the sole carbon source (MM-Glc), followed by incubation at 37°C for 20 h. One-milliliter samples then were centrifuged and the bacterial pellets were gently resuspended in MM containing no carbohydrate (MM-NC). These suspensions were diluted 1:50 in MM-NC before being used to inoculate MM containing 0.5% carbohydrate. Growth experiments were performed in replicates of 6 (laminarin, yeast β-glucan, barley β-glucan, and curdlan) or 3 (glucose and H_2_O) in 96-well plates. Growth was monitored by measuring the *A*_600_, and data were processed using Gen5 software (BioTek). Growth was quantified in each assay by first identifying a minimum time point (*A*_min_) at which the *A*_600_ had increased by 10% over a baseline reading taken during the first 500 min of incubation. We then identified the time point at which the *A*_600_ reached its maximum (*A*_max_) immediately after exponential growth. The growth rate for each well was defined by (*A*_max_ − *A*_min_)/(*T*_max_ − *T*_min_), where *T*_max_ and *T*_min_ are the corresponding time values for each absorbance. Cultures where the density did not increase by at least 0.1 (*A*_600_) were considered to have no growth.

### Quantitative reverse transcription-PCR (qRT-PCR).

B. uniformis was capable of growth on all carbon sources of interest in this study and, therefore, was cultured directly in 3 ml of MM containing 0.5% (wt/vol) carbohydrate, as described above. B. thetaiotaomicron and *B. fluxus* were precultured on MM-Glc, pelleted, washed, resuspended twice in MM-NC, and inoculated to an *A*_600_ of ∼0.3 in 4 ml of MM containing 0.5% (wt/vol) carbohydrate. Bacterial cultures were harvested in triplicate (at mid-log phase [*A*_600_, ∼0.6] for B. uniformis and after 5 h of incubation for B. thetaiotaomicron and B. fluxus), placed in RNA protect (Qiagen) for immediate stabilization of RNA, and then stored at –20°C. RNA was extracted and purified with the RNeasy minikit (Qiagen), and RNA purity was assessed by spectrophotometry. One μg of RNA was used for reverse transcription and synthesis of the cDNA (SuperScript VILO master mix; Invitrogen). Quantitative PCRs (20 μl final volume) using specific primers were performed with a SensiFast SYBR Lo-ROX kit (Bioline) on a 7500 Fast real-time PCR system (Applied Biosystems) (see Table S9 at https://doi.org/10.14288/1.0388792). Data were normalized to 16S rRNA transcript levels, and changes in expression levels were calculated as fold change compared with levels for cultures of MM containing glucose.

### Bioinformatics, gene cloning, and site-directed mutagenesis.

Potential 1,3GULs were identified by homology searches of sequences available in the Joint Genome Institute Integrated Microbial Genomes and Metagenomes (JGI-IMG/M) Database (65) and other sources (41). Signal peptides and subcellular localization were predicted by protein sequence analysis (66–68). The gene fragments corresponding to BACUNI_01484 to BACUNI_01488 (encoding *Bu*GH3, *Bu*GH158, *Bu*GH16, *Bu*SGBP-B, and *Bu*SGBP-A), H207DRAFT_02225 to H207DRAFT_02227 (encoding *Bt*SGBP-A, *Bt*SGBP-B, and *Bt*GH16), HMPREF9446_00612 to HMPREF9446_00614 (encoding *Bf*GH158, *Bf*SGBP-B, and *Bf*SGBP-A), and HMPREF1991_02176 (encoding *Pl*GH16) were PCR amplified from genomic DNA using the Q5 high-fidelity polymerase (NEB) with primers designed to exclude signal peptides and lipidation cysteines (66, 69) (Table S10 at the URL mentioned above). The PCR introduced SalI/XhoI (NEB) restriction sites to the flanks of BACUNI_01484 and _01486 target genes, and the amplified DNA products were ligated into the expression vector pET28a such that the encoded recombinant proteins contain an N-terminal His_6_ tag. The rest of the PCR products contained appropriate pMCSG complementary sequences for subsequent ligation-ndependent cloning into pMCSG53 or pMCSG-GST plasmids, providing an N-terminal His_6_ tag or an N-terminal His_6_-glutathione *S*-transferase (GST) tag (70). Successful cloning was confirmed by colony PCR (GoTaq polymerase from Promega) and sequencing (Genewiz). The site-directed mutants (71) *Bu*GH158 N136A, E137A, and E220A were generated using pMCSG53::*Bu*GH158 as a template DNA (Table S11 at the URL mentioned above).

### Recombinant protein production and purification.

Recombinant proteins were produced in Escherichia coli BL21(DE3) cells cultured in TB broth containing ampicillin (50 μg·ml^−1^) or kanamycin (50 μg·ml^−1^) at 37°C (200 rpm). Cells were grown to mid-exponential phase (optical density at 600 nm [OD_600_], ∼0.4 to 0.6). Overexpression was induced by adding isopropyl β-d-thiogalactopyranoside (IPTG) to a final concentration of 0.5 mM, and the cultures were further grown at 16°C (200 rpm) for 18 h. The cells were harvested by centrifugation and sonicated, and His_6_-tagged recombinant proteins were purified via immobilized nickel affinity chromatography (His-Trap; GE Healthcare) utilizing a gradient elution of up to 100% elution buffer containing 20 mM sodium phosphate, pH 7.4, 500 mM NaCl, and 500 mM imidazole in a BioLogic fast protein liquid chromatography system (Bio-Rad). The purity of the recombinant proteins was determined by SDS-PAGE (Fig. S15 and S16 at https://doi.org/10.14288/1.0388792), and their concentrations were determined from calculated molar extinction coefficients at 280 nm using an Epoch microplate spectrophotometer (BioTek).

Selenomethionine-labeled protein was produced by inhibition of methionine biosynthesis in E. coli BL21(DE3) (72). Briefly, cells were grown in 1 liter of M9 minimal medium supplemented with 100 μg ml^−1^ ampicillin at 37°C with shaking until the OD_600_ reached 0.6. At this point, 100 mg each of l-lysine, l-threonine, and l-phenylalanine and 50 mg each of l-leucine, l-isoleucine, l-valine, and l-selenomethionine were added to the medium and shaken for a further 15 min before inducing expression with 0.5 mM IPTG. The culture was transferred to 16°C and incubated for an additional 24 h. Nickel affinity chromatography was conducted as described above using HEPES buffer instead of sodium phosphate. Native and selenomethionine-labeled proteins for crystallography were further purified by size exclusion chromatography through a Superdex 75 resin (GE Healthcare Life Sciences) in an XK 16/100 column (GE Healthcare Life Sciences) run in 10 mM HEPES, pH 7.0, at 0.8 ml min^−1^.

### Affinity gel electrophoresis.

Affinity polyacrylamide gel electrophoresis was performed for 180 min at 80 V and room temperature on nondenaturing 10% (wt/vol) polyacrylamide gels containing a polysaccharide concentration of 0.1% (wt/vol) (or water for a control), essentially as previously described (15, 26, 46). Five micrograms of the tested SGBP proteins, along with bovine serum albumin (BSA) used as noninteracting negative-control protein, were loaded on the gels.

### ITC.

Isothermal titration calorimetry (ITC) was performed using the MicroCal VP-ITC titration calorimeter equilibrated to 25°C, essentially as previously described (15, 26, 46). The proteins (20 to 100 μM) were placed in the sample cell, and the syringe was loaded with 2.5 mg/ml polysaccharide or 0.5 to 2 mM oligosaccharide. Following an initial injection of 2 μl, 25 subsequent injections of 10 μl were performed with stirring at 280 rpm, and the resulting heat of the reaction was recorded. Integrated heats were fit to a single-site model using Microcal Origin v7.0 to derive *n*, *K*_a_, and Δ*H* values.

### Carbohydrate analytical method.

High-performance anion-exchange chromatography with pulsed amperometric detection (HPAEC-PAD) was performed on Carbopac PA200 guard and analytical columns connected in series on a Dionex ICS-5000 high-performance liquid chromatography system, operated by Chromeleon software, version 7 (Thermo Scientific), essentially as previously described (73). Solvent A was ultrapure water, solvent B was 1 M sodium hydroxide, and solvent C was 1 M sodium acetate (anhydrous bio ultra-grade; Sigma-Aldrich). The injection volume was 10 μl, and the gradient was the following: 0 to 5 min, 10% B and 3.5% C; 5 to 12 min, 10% B, linear gradient from 3.5 to 30% C; 12.0 to 12.1 min, 50% B, 50% C; 12.1 to 13.0 min, exponential gradient (curve setting, 9) of B and C back to initial conditions; 13 to 17 min, initial conditions.

### Enzymatic assays.

Polysaccharide hydrolysis was quantified using a BCA reducing-sugar assay (74). Assays were conducted in a final volume of 100 μl at the optimum pH and 37°C for 10 min. Reactions were terminated by the addition of an equal volume (100 μl) of BCA reagent. Color was developed by heating to 80°C for 20 min before reading *A*_563_. Glucose (25 to 150 μM) was used to generate a standard curve for quantitation. The pH and temperature optima of each enzyme were initially determined using the same enzyme reaction assay to quantify reducing ends over 10 min of incubation with 1.0 mg/ml laminarin in different buffers at 50 mM: sodium citrate (pH 3.0 to 6.5), sodium phosphate (pH 6.5 to 8.0), and glycine (pH 9.0 to 10.5). To determine Michaelis-Menten parameters, different concentrations of polysaccharide solutions were used over the range of 0.025 to 3 mg·ml^−1^ with the appropriate concentration of enzyme for 10 min, and the numbers of reducing ends released were quantified as described above.

The release of glucose from oligosaccharides was quantified using the d-glucose HK assay kit from Megazyme (Bray, Ireland), modified for use as a continuous assay exactly as previously described (19).

To measure enzyme activity on chromogenic glycosides, the release of *para*-nitrophenyl was monitored by following the *A*_405_ in a 1-cm-path-length quartz cuvette with a Cary 60 UV-visible spectrophotometer (Agilent Technologies). Reaction mixtures in 250 μl at the optimum pH and 37°C were assayed with nine different substrate concentrations, and the rate was calculated using an extinction coefficient determined according to the buffer used. Endpoint assays were used for pH and temperature optima (same range as that described above) of *Bu*GH3. Reactions were terminated after 10 min by the addition of 100 μl of 1 M Na_2_CO_3_ to raise the pH, and the *A*_405_ was measured in 96-well plates on an Epoch microplate spectrophotometer (BioTek). An extinction coefficient of 18,100 M^−1 ^cm^−1^ was used for these assays. Continuous assays were used for initial-rate saturation kinetics and reactions, initiated by adding 25 μl of enzyme solution to 225 μl of the remaining assay mixture in the optimum pH buffer at 37°C.

### Crystallization and structure determination.

Initial sitting-drop crystal screens were set up in 96-well plates using a Phoenix robot (Art Robbin Instruments) and were stored at room temperature. A hit was obtained in pHClear (Qiagen), condition E12 (0.1 M bicine, pH 9.0, 1.6 M ammonium sulfate), with purified *Bu*GH158 at 22.7 mg ml^−1^ and optimized in larger hanging drops in a grid screen by varying pH and ammonium sulfate concentrations in 24-well plates. Crystals were cryoprotected in crystallization solution supplemented with 2 M lithium acetate before flash freezing with liquid nitrogen, and diffraction data were collected at the Advanced Photon Source (APS) beamline 23ID-D. Selenomethionine-labeled crystals were obtained in the same grid screen as the native crystals but required macroseeding with fine needles to obtain crystals of sufficient thickness. Crystals were cryoprotected in crystallization solution supplemented with 4 M lithium chloride before flash freezing with liquid nitrogen. Diffraction data were collected at the Stanford Synchrotron Radiation Lightsource (SSRL) beamline 9-2 at the selenium absorption peak, inflection, and high-energy remote wavelengths as determined by a fluorescence scan. Data sets were indexed and integrated with XDS (75), space groups were determined with Pointless (76), and data reduction was performed with Aimless (77). Phasing by multiple anomalous dispersion, density modification, and initial model building was performed in AutoSol (78) in the Phenix suite (79). This selenomethionine-labeled structure was used as the search model for molecular replacement with the native crystal data using Phaser (80). After initial refinement in Phenix.refine (81), iterative rounds of manual model building and refinement were conducted with Coot (82) and Refmac5 (83), respectively, in the CCP4i2 suite (84). The quality of the model was monitored throughout using Molprobity (85). A structure similarity search was conducted using the Dali server (86).

### ^1^H-NMR determination of catalytic mechanism.

*Bu*GH158 in 50 mM sodium phosphate, pH 7.0, and laminaribiose-β-CNP were independently lyophilized and resuspended in 99.9% D_2_O. After recording an initial ^1^H-NMR spectrum of the substrate (Bruker Avance 400-MHz spectrometer), the enzyme was added to obtain final concentrations of 20 μM *Bu*GH158 and 10 mM laminaribiose-β-CNP. Spectra were recorded at appropriate time intervals thereafter to observe the first-formed product anomer and subsequent mutarotation.

### Survey of human metagenomic data sets.

Available cohorts of human gut metagenomic sequence data (National Center for Biotechnology Information projects PRJNA422434 [87], PRJEB10878 [88], PRJEB12123 [89], PRJEB12124 [90], PRJEB15371 [91], PRJEB6997 [92], PRJDB3601 [93], PRJNA48479 [94], PRJEB4336 [95], PRJEB2054 [96], PRJNA392180 [51], and PRJNA527208 [97]) were searched for the presence of 1,3GUL nucleotide sequences from *B. fluxus* (12.5 kb), B. thetaiotaomicron (12.6 kb), and B. uniformis (14.5 kb) using the following workflow. Each 1,3GUL nucleotide sequence was used separately as a template, and then magic-blast v1.5.0 (98) was used to recruit raw Illumina reads from the available metagenomic data sets with an identity cutoff of 97%. The alignment files next were used to generate a coverage map using bedtools v2.29.0 (99) to calculate the percent coverage of each sample against each reference. We considered a metagenomic data sample to be positive for a particular 1,3GUL if it had at least 70% of the corresponding 1,3GUL nucleotide sequence covered (since the three 1,3GUL sequences were very similar in size, no normalization was made for PUL template variation).

### Data availability.

The crystal structure data sets generated (coordinate files and structure factors) have been deposited in the Protein Data Bank (PDB) under the accession code 6PAL. We declare that the data supporting the findings of this study are available within the article and the supplemental material, the latter of which is available at https://doi.org/10.14288/1.0388792.
